# Supplementary material for: Oxidative stress and GPX2 control pancreatic vs. non-pancreatic cell fate in human endoderm
Source: Nat Commun. 2026 Jan 3;17:1407. doi: 10.1038/s41467-025-68145-x (PMC12881361; doi:10.1038/s41467-025-68145-x)
Supplement: Supplementary file 1 — Supplementary Information [file 41467_2025_68145_MOESM1_ESM.pdf]

Oxidative stress and GPX2 control pancreatic vs. non-pancreatic cell fate in human endoderm

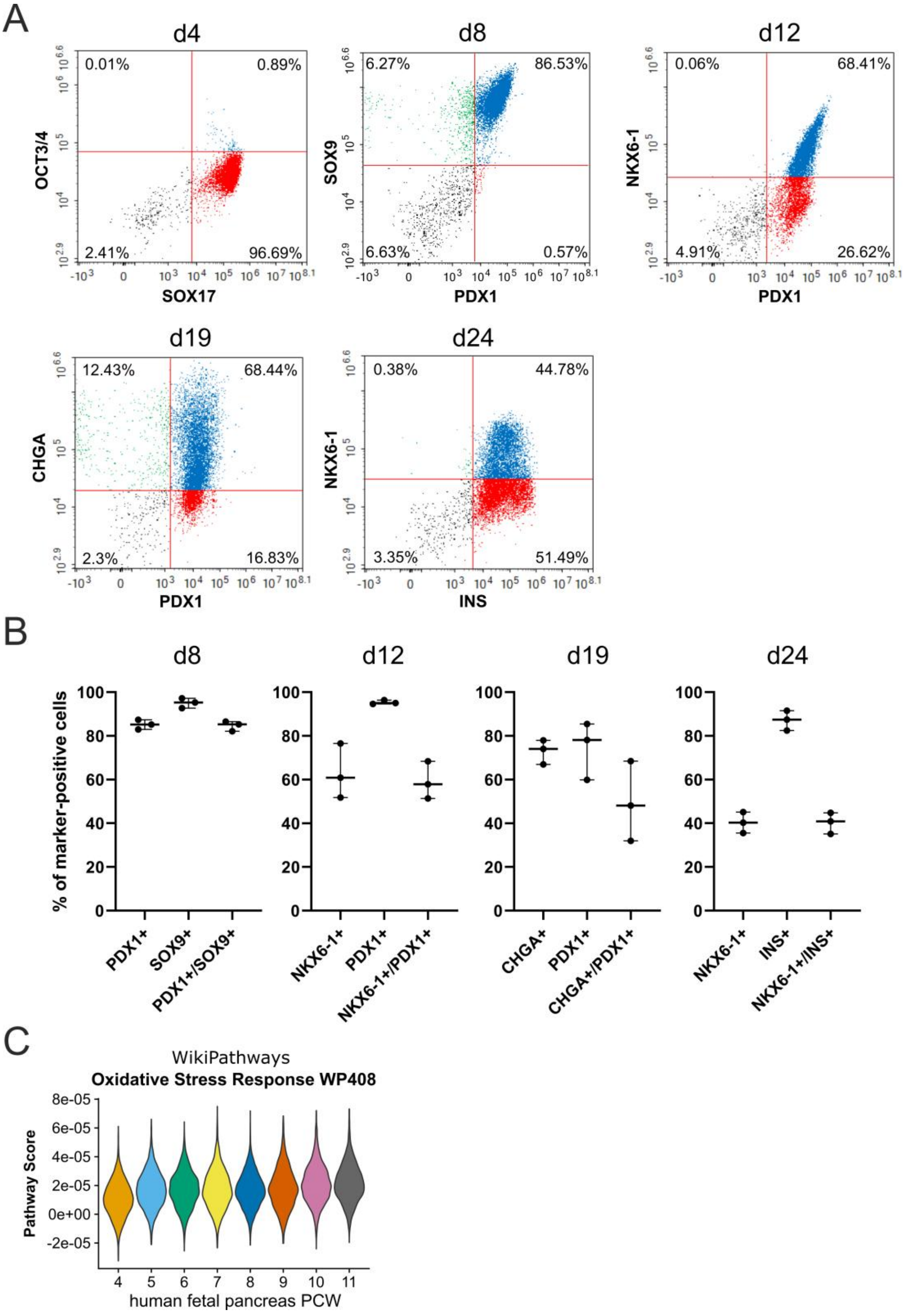

Supplementary Figure 1. Oxidative stress response increases during human fetal pancreas development

A. Representative flow cytometry analysis of β cell differentiation efficiency in selected timepoints: d4 (DE stage; OCT3/4 [FITC-H] and SOX17 [APC-H]), d8 (PEN stage; SOX9 [FITC-H] and PDX1 [APC-H]), d12 (PP stage; NKX6-1 [FITC-H] and PDX1 [APC-H]), d19 (late EP; CHGA[FITC-H] and PDX1 [APC-H]), and d24 (SC-β stage; NKX6-1 [FITC-H] and INS [APC-H]).
B. Quantification of flow cytometry data during pancreatic differentiation at key stages: pancreatic endoderm (PEN, day 8), pancreatic progenitor (PP, day 12), late endocrine progenitor (late EP, day 19), and SC-β cell stage (day 24). Expression and co-expression of stage-specific markers were analyzed: PDX1 and SOX9 at day 8; NKX6-1 and PDX1 at day 12; CHGA and PDX1 at day 19; and NKX6-1 and INS at day 24. Each dot represents one experiment, with mean and 95% confidence intervals (CIs) indicated. $N = 3$ independent experiments. Source data are provided as a Source Data file.
C. Violin plots show Oxidative Stress Response (WP408, WikiPathways) scores distribution for PCW4-11 human fetal pancreatic epithelial and endocrine cells (scRNA-seq data from Ma *et* al., 2023[1]).

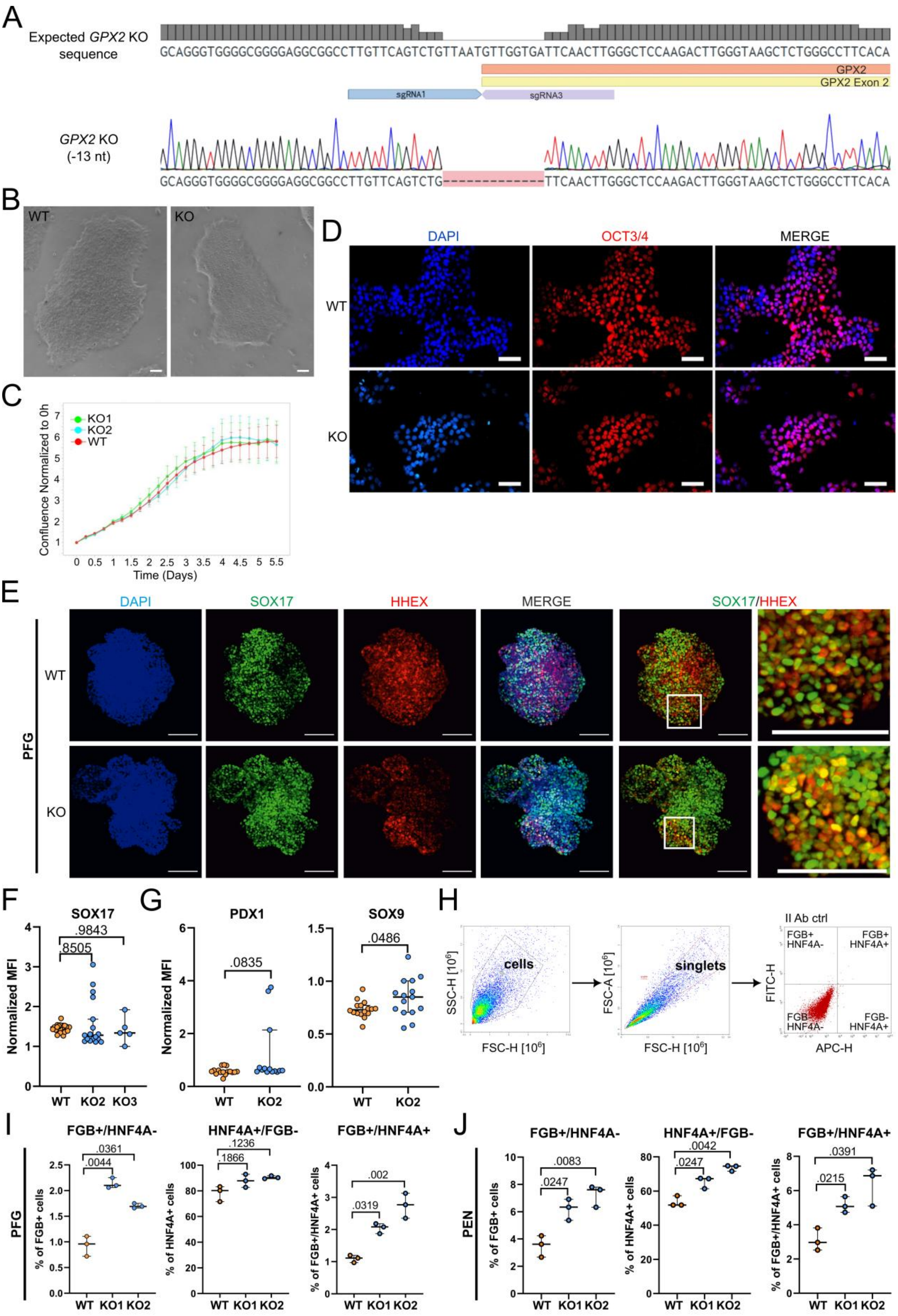

A
Expected GPX2 KO sequence
GCAGGGTGGGGCGGGGAGGCGGCCTTGTTCAGTCTGTTAATGTTGGTGATTCAACTTGGGCTCCAAGACTTGGGTAAGCTCTGGGCCTTCACA
GPX2
GPX2 Exon 2
sgRNA1
sgRNA3
GPX2 KO (-13 nt)
GCAGGGTGGGGCGGGGAGGCGGCCTTGTTCAGTCTG-------------TTCAACTTGGGCTCCAAGACTTGGGTAAGCTCTGGGCCTTCACA
B
WT
KO
C
KO1
KO2
WT
Confluence Normalized to 0h
Time (Days)
D
DAPI
OCT3/4
MERGE
WT
KO
E
DAPI
SOX17
HHEX
MERGE
SOX17/HHEX
WT
KO
PFG
F
SOX17
.9843
.8505
Normalized MFI
WT
KO2
KO3
G
PDX1
.0835
Normalized MFI
WT
KO2
SOX9
.0486
WT
KO2
H
SSC-H [10^6]
cells
FSC-H [10^6]
FSC-A [10^6]
singlets
FSC-H [10^6]
II Ab ctrl
FGB+ HNF4A-
FGB+ HNF4A+
FGB- HNF4A-
FGB- HNF4A+
FITC-H
APC-H
I
PFG
FGB+/HNF4A-
.0361
.0044
% of FGB+ cells
WT KO1 KO2
HNF4A+/FGB-
.1236
.1866
% of HNF4A+ cells
WT KO1 KO2
FGB+/HNF4A+
.002
.0319
% of FGB+/HNF4A+ cells
WT KO1 KO2
J
PEN
FGB+/HNF4A-
.0083
.0247
% of FGB+ cells
WT KO1 KO2
HNF4A+/FGB-
.0042
.0247
% of HNF4A+ cells
WT KO1 KO2
FGB+/HNF4A+
.0391
.0215
% of FGB+/HNF4A+ cells
WT KO1 KO2

Supplementary Figure 2. Validation of *GPX2* KO hPSCs

A. Chromatogram shows DNA sequencing results of *GPX2* KO and the reference sequence of WT cells. The expected sequence for *GPX2* KO is marked by gray blocks. No substitutions or deletions were observed within the flanking sequence. The locations of sgRNA1 and sgRNA3, which flank the *GPX2* coding sequence, are shown. A 13-nt deletion is highlighted with a red box on the chromatogram.

B. Representative bright field images of WT and KO hPSC colonies. Scale bar = 100 μm.

C. Representative growth curves of WT (red), KO1 (green) and KO2 (blue) hPSCs monitored by live imaging during a 5.5-day period. Confluence values, normalized to the starting time point, showed no significant differences between WT and KO hPSCs. WT is marked in red, KO1 in green and KO2 in blue. For each point SEM is indicated. N = 3 independent experiments. Source data are provided as a Source Data file.

D. WT and KO hPSCs stained for pluripotency marker OCT3/4 (red). DAPI marks nuclei (blue). Scale bar = 100 μm.

E. WT and KO spheroids at the PFG stage were stained for SOX17 (green) and HHEX (red) marker genes. DAPI marks nuclei (blue). Scale bar = 100 μm. N = 3 independent experiments. Magnified views of SOX17/HHEX co-expression are shown.

F. Dot plot shows quantification of SOX17 mean fluorescence intensity (MFI) relative to DAPI for WT (orange), KO2, and KO3 (blue) spheroids at the DE stage. Each dot represents one image, with median and 95% confidence intervals (CIs) indicated. N = 3 independent experiments. An unpaired, two-sided t-test was used to determine statistical significance. Source data are provided as a Source Data file.

G. Dot plot shows quantification of the mean fluorescence intensity (MFI) of PDX1 and SOX9 relative to DAPI for WT (orange) and KO2 (blue) spheroids at the PEN stage. Each dot represents one image. The median with 95% CIs is shown on the graph. The *p*-values were calculated using an unpaired, two-sided t-test. N = 3 independent experiments. Source data are provided as a Source Data file.

H. Gating strategy for identification of cells co-expressing HNF4A and FGB at day 6 and 8 of differentiation. Cells were gated based on SSC-H vs. FSC-H. Next, single cells (singlets) were selected using FSC-A vs. FSC-H and subsequently $HNF4A^+$ and FGB^+ cells were selected using FITC-H (FGB) vs. APC-H (HNF4A).

I. Flow cytometry-based quantification of FGB^+ and $HNF4A^+$ cells, as well as their co-expression, at the PFG stage. WT cells are shown in orange, and *GPX2* KO (clonal lines KO1 and KO2) cells are in blue. Each dot represents one experiment, with median and 95%

confidence intervals (CIs) indicated. $N = 3$ biological repeats. Source data are provided as a Source Data file.

J. Flow cytometry-based quantification of FGB^+ and $HNF4A^+$ cells, as well as their co-expression, at the PEN stage. WT cells are shown in orange, and *GPX2* KO (clonal lines KO1 and KO2) cells are marked in blue. Each dot represents one experiment, with median and 95% confidence intervals (CIs) indicated. $N = 3$ independent experiments. Source data are provided as a Source Data file.

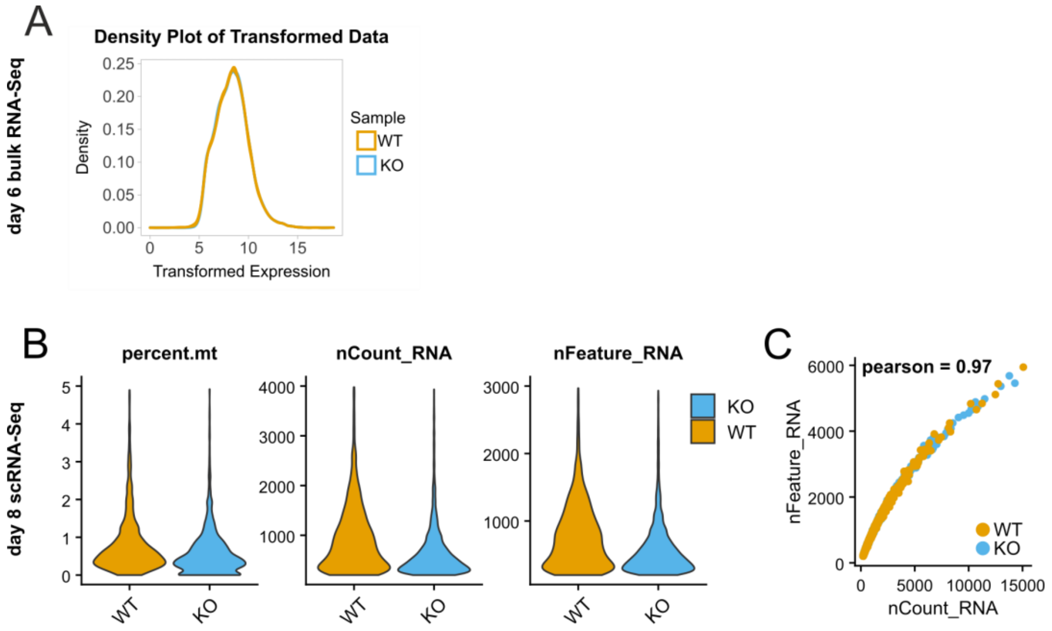

Supplementary Figure 3. Quality control metrics for RNA sequencing at PFG and PEN stages

A. Density plot shows the distribution of transformed expression values for WT (orange) and KO (blue) from RNA-Seq data at the PEN stage on day 6 of differentiation.

B. Violin plots represent quality control (QC) metrics for scRNA-Seq data at day 8 of differentiation for WT (orange) and KO (clonal line KO1, blue) cells at the PFG stage on day 6 of differentiation. Percent.mt - the percentage of reads mapped to the mitochondrial genome, nCount_RNA - the total number of transcripts identified in a cell, nFeature_RNA - the count of distinct genes detected in each cell.

C. Pearson correlation plot depicts the association between total transcript counts (nCount_RNA) and the number of expressed genes (nFeature_RNA) in single-cell RNA-Seq data for WT (orange) and KO (blue) cells at the PFG stage on day 8 of differentiation.

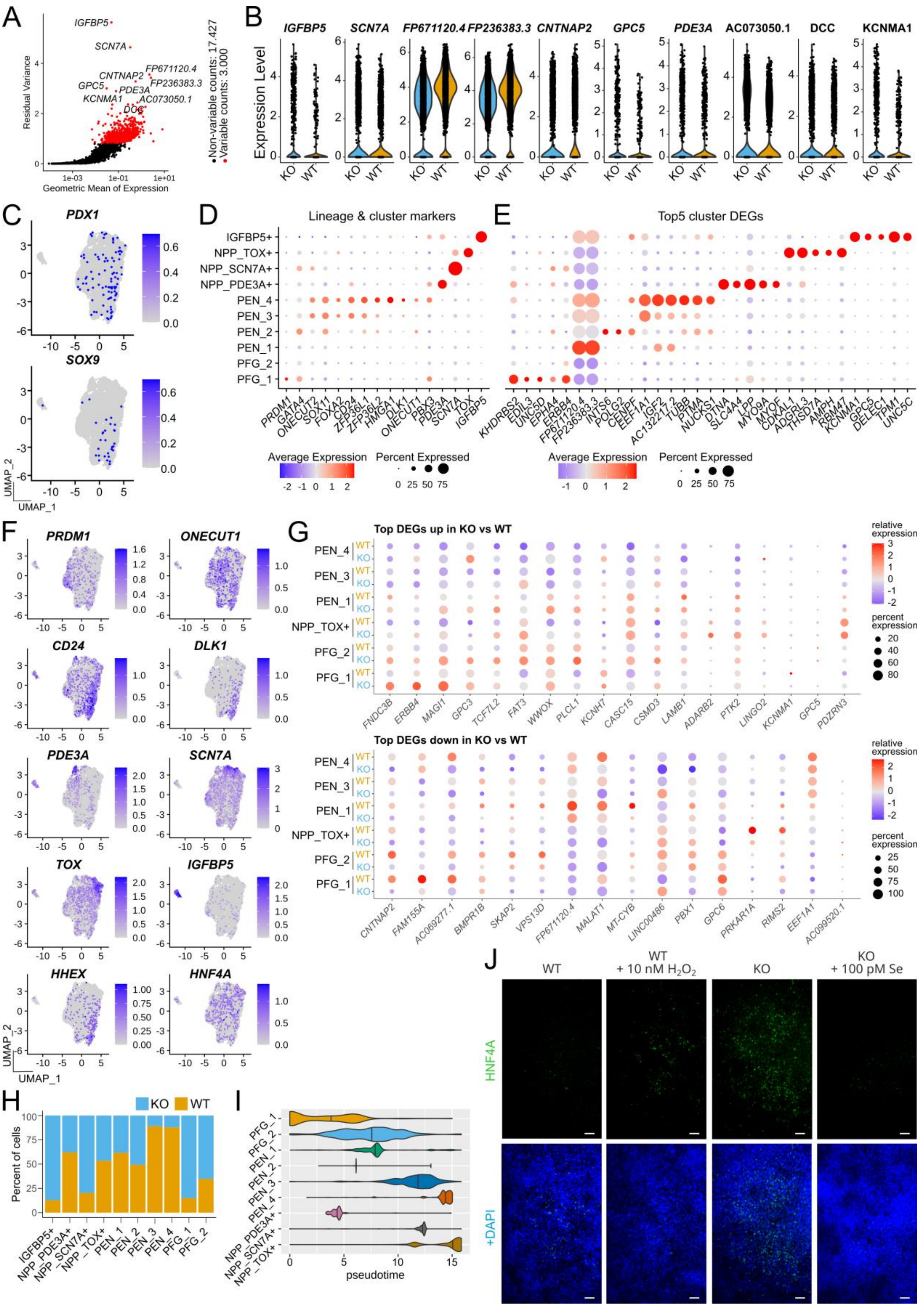

Supplementary Figure 4. Transcriptional heterogeneity of WT and KO PFG at the single-cell level

A. Plot represents highly variable features on day 8 of differentiation. A total of 3,000 variable features is highlighted in red, while 11,427 non-variable features are displayed in black. Each dot represents one gene.

B. Violin plots show differences between WT (orange) and KO (clonal line KO1, blue) in the expression level of the top 10 highly variable features. Each dot represents a single cell.

C. UMAP shows the expression level of PDX1 (upper panel) and SOX9 (lower panel) in PEN cells, based on scRNA-Seq results from day 8 of differentiation. Cells that express PDX1 or SOX9 are marked in blue.

D. Marker genes for each of the 10 clusters are shown. Cell identities were assigned based on these marker genes. Dot color indicates average expression (red = high), dot size indicates fraction of cells expressing each gene. Source data are provided.

E. Dot plot shows the top 5 differentially expressed genes for each of the 10 clusters of scRNA-Seq from day 8 of differentiation. High average gene expression is indicated in red. Dot size represents the percentage of cells expressing selected markers. Source data are provided as a Source Data file.

F. UMAP shows the expression level of marker genes characteristic for 10 clusters. Cells that contain highly expressed marker genes are marked in blue.

G. Top 18 up-regulated (upper) and 16 down-regulated (lower) genes in KO1 vs. WT at differentiation day 8. Red = high expression, blue = low; dot size = percent of cells expressing each gene Source data are provided as a Source Data file.

H. Bar plot represents the proportion of each cluster by genotype (WT - orange, KO - blue).

I. Violin plot represents the pseudotime distribution of each cluster of cells at the PEN stage.

J. Representative IF for HNF4A protein (green) in untreated WT, KO (clonal line KO2) and treated: WT+ H_2O_2 and KO+Se cells. DAPI marks nuclei in blue. Scale bar = 200 μm.

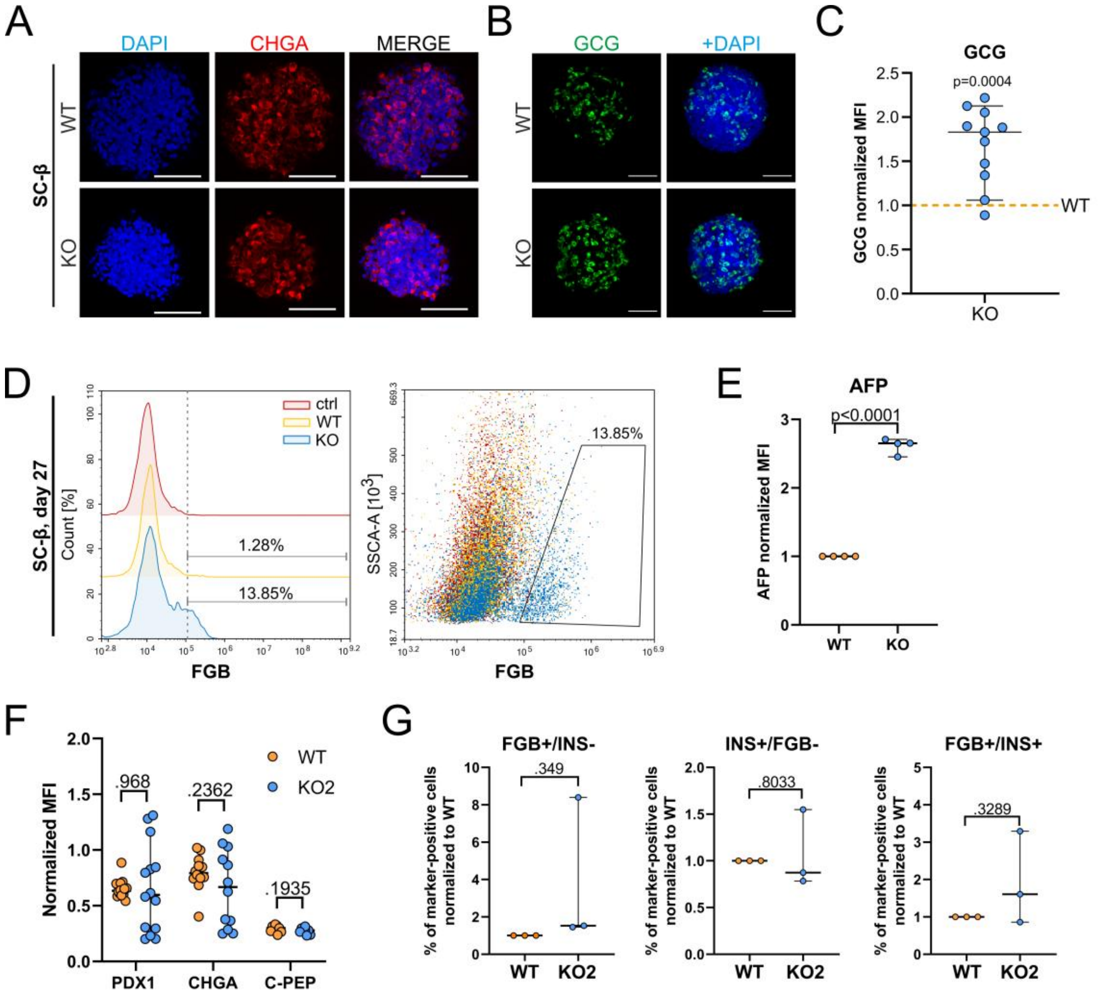

Supplementary Figure 5. Changes in selected protein marker levels in *GPX2*-deficient cells

A. WT and KO (clonal line KO1) spheroids on the SC-β cell stage were stained for endocrine cell marker, chromogranin A, CHGA (red). DAPI marks nuclei (blue). Scale bar = 100 µm. $N = 3$ independent experiments.

B. WT and KO (clonal line KO1) spheroids on the SC-β cell stage were stained for glucagon, GCG (green), α cell marker. DAPI marks nuclei (blue). Scale bar = 100 µm. $N = 3$ independent experiments.

C. Dot plot shows IF quantifications of GCG immunofluorescence signal. Each dot represents one image, with median and 95% confidence intervals (CIs) indicated. $N = 3$ independent experiments. An unpaired, two-sided t-test was used to determine statistical significance. Source data are provided as a Source Data file.

D. FGB expression was measured using flow cytometry, results are on a histogram and scatter plot. WT and KO (clonal line KO1) cells are presented in orange and blue, respectively. Control

cells (stained only with secondary antibody) are marked in red. The percentage of cells that express FGB protein is also displayed. *N* = 2 biological repeats.

E. Dot plot represents the quantification of mean fluorescence intensity (MFI) of AFP relative to DAPI for WT (orange) and KO (clonal line KO1, blue) cells at the SC-β cell stage. Data are presented as mean on the plot; error bars are 95% CIs. The *p*-values were calculated using an unpaired, two-sided t-test. *N* = 4 biological repeats. Source data are provided as a Source Data file.

F. Quantification of the mean fluorescence intensity (MFI) of PDX1 and SOX9 relative to DAPI for WT (orange) and KO2 (blue) spheroids at SC-β cell stage is shown as a dot plot. Each dot represents one image, with mean and 95% confidence intervals (CIs) indicated. *N* = 3 independent experiments. An unpaired, two-sided t-test was used to determine statistical significance. Source data are provided as a Source Data file.

G. Flow cytometry-based quantification of FGB^+ and INS^+ cells, as well their co-expression, at the SC-β cell stage. WT cells are shown in orange, and *GPX2* knockout (KO2) cells in blue. Each dot represents one experiment. An unpaired, two-sided t-test was used to determine statistical significance.with median and 95% confidence intervals (CIs) indicated. *N* = 3 independent experiments. Source data are provided as a Source Data file.

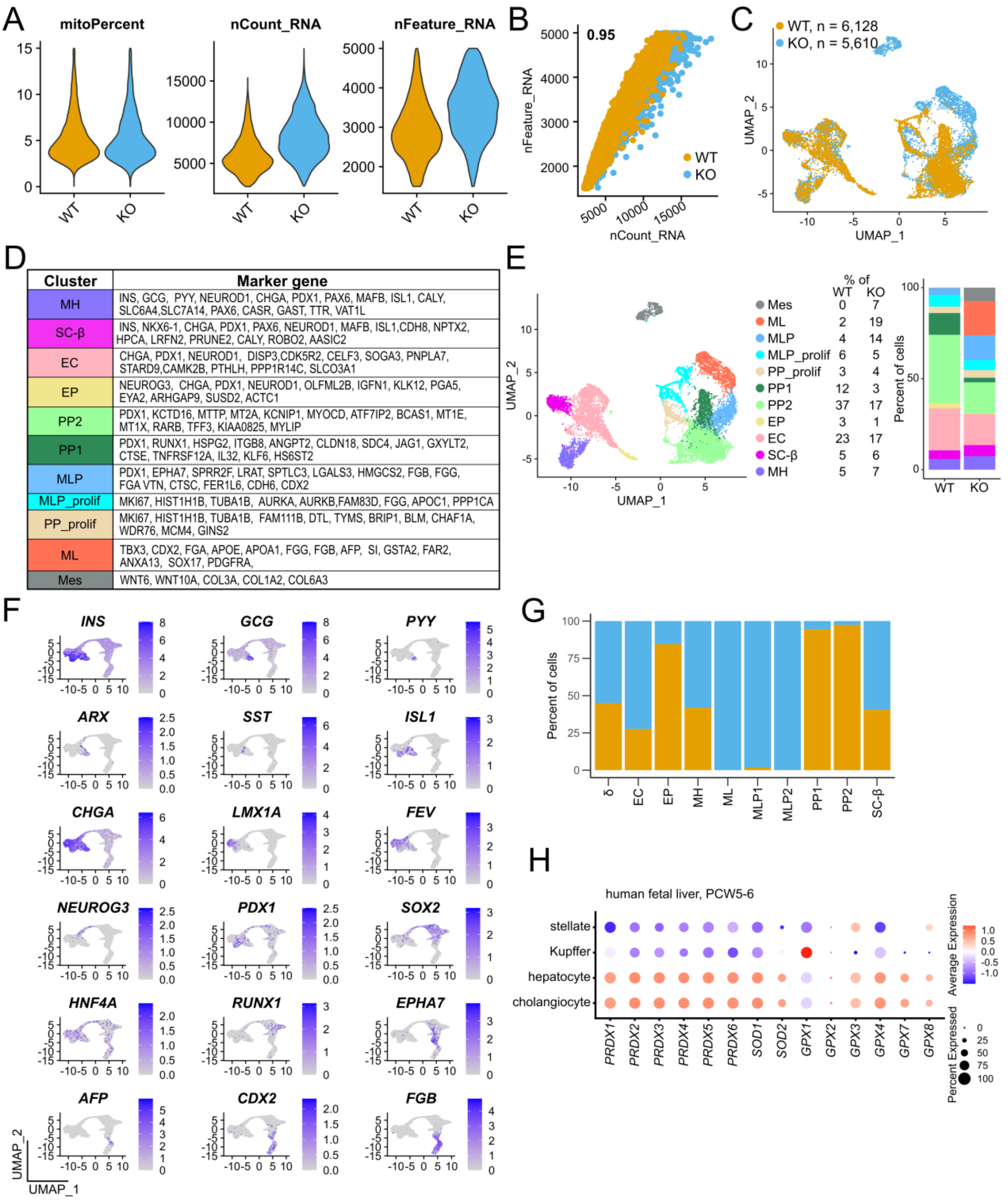

Supplementary Figure 6. Molecular heterogeneity of WT and KO cells at early SC-β-cell stage of pancreatic differentiation

A. Violin plots represent quality control (QC) metrics for scRNA-Seq data at day 21 of differentiation for WT (orange) and KO (clonal line KO1, blue). mitoPercent - the percentage of reads mapped to the mitochondrial genome, nCount_RNA - the total number of molecules identified in a cell, nFeature_RNA - the count of distinct genes detected in each cell.

B. Pearson correlation plot depicts the association between total transcript counts (nCount_RNA) and the number of expressed genes (nFeature_RNA) in single-cell RNA-Seq data for WT (orange) and KO (clonal line KO1, blue) cells at the SC-β stage on day 21 of differentiation.

C. UMAP embedding projection of an integrated dataset of 11,738 SC-β cells, sequenced on day 21 of differentiation. Cells are labeled by sample origin (WT – orange, KO – blue). Each dot represents a single cell. 6,128 and 5,610 cells were sequenced for WT and KO, respectively.

D. Table shows marker genes specific for each cluster. Clusters are colored according to the scRNA-Seq UMAP representation shown in (E).

E. Clustering of datasets from (C) into various cell types at the SC-β cell stage, organized into 11 distinct clusters, visualized using a UMAP projection. Each dot represents a single cell, with cell types color-coded. The percentage representation of each cluster in WT and KO genotypes is shown numerically and displayed in a bar plot, with colors corresponding to those observed on UMAP.

F. UMAP shows expression levels of 18 marker genes characteristic for clusters. Cells that contain highly expressed marker genes are marked in blue.

G. Bar plot represents the proportion of each cluster by genotype (WT – orange, KO – blue).

H. Oxidative stress response genes expression in early PCW5-6 human liver. The higher average gene expression is shown in red, and the lower expression in blue.

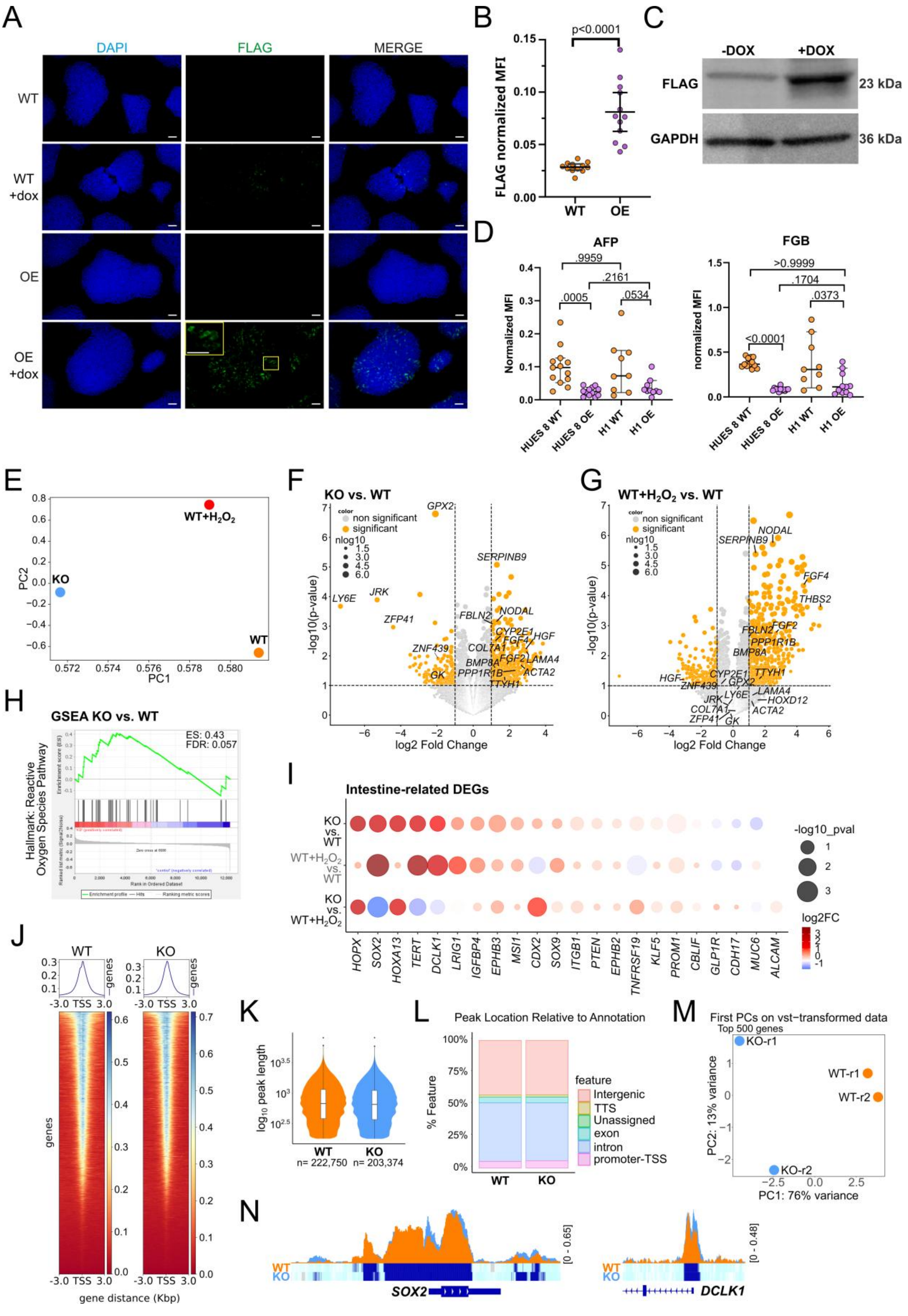

A
DAPI
FLAG
MERGE
WT
WT +dox
OE
OE +dox
B
p<0.0001
FLAG normalized MFI
WT
OE
C
-DOX
+DOX
FLAG
GAPDH
23 kDa
36 kDa
D
AFP
FGB
Normalized MFI
normalized MFI
HUES 8 WT
HUES 8 OE
H1 WT
H1 OE
E
WT+H2O2
KO
WT
PC1
PC2
F
KO vs. WT
G
WT+H2O2 vs. WT
log2 Fold Change
-log10(p-value)
non significant
significant
H
GSEA KO vs. WT
Hallmark: Reactive Oxygen Species Pathway
ES: 0.43
FDR: 0.057
I
Intestine-related DEGs
KO vs. WT
WT+H2O2 vs. WT
KO vs. WT+H2O2
-log10_pval
log2FC
J
gene distance (Kbp)
K
log10 peak length
n= 222,750
n= 203,374
L
Peak Location Relative to Annotation
% Feature
feature
Intergenic
TTS
Unassigned
exon
intron
promoter-TSS
M
First PCs on vst-transformed data
Top 500 genes
KO-r1
KO-r2
WT-r1
WT-r2
PC1: 76% variance
PC2: 13% variance
N
SOX2
DCLK1
[0 - 0.65]
[0 - 0.48]

Supplementary Figure 7. Inducible GPX2 overexpression system and spontaneous differentiation of DE cells with altered GPX2 expression and oxidative stress manipulation

A. WT and OE hPSCs (HUES8 background) stained for FLAG (green). Doxycycline (1 μg/mL) was added to OE cells to induce GPX2 overexpression, and to WT cells as a vehicle control. DAPI marks nuclei (blue). Scale bar = 200 μm. *N* = 3 independent experiments.

B. Dot plot shows IF quantifications of FLAG immunofluorescence signal. Each dot represents one image, with median and 95% confidence intervals (CIs) indicated. *N* = 3 independent experiments. An unpaired, two-sided t-test was used to determine statistical significance. WT is shown in orange, and OE in violet. Source data are provided as a Source Data file.

C. Western blot shows an increase in FLAG levels in OE hPSCs (HUES8 background) upon doxycycline treatment, confirming the upregulation of GPX2, which carries a FLAG tag. GAPDH was used as a loading control. Source data are provided as a Source Data file.

D. Dot plot shows IF quantifications of AFP and FGB mean fluorescent intensity (MFI) in HUES 8 and H1 hESC lines overexpressing GPX2 protein. Each dot represents one image, with median and 95% confidence intervals (CIs) indicated. *N* = 3 independent experiments. An unpaired, two-sided t-test was used to determine statistical significance. WT is shown in orange, and OE in violet. Source data are provided as a Source Data file.

E. PCA analysis shows transcriptional profiles of WT (orange), WT+H_2O_2 (red) and KO (clonal line KO1, blue) cells differentiated spontaneously until day 8.

F. Volcano plot illustrates downregulated and upregulated DEGs in KO (clonal line KO1) cells differentiated spontaneously until day 8 compared to their WT counterparts. Genes with statistically significant expression changes between conditions are marked in yellow. Selected genes are labeled on the plot. Source data are provided as a Source Data file.

G. Volcano plot illustrates downregulated and upregulated DEGs in WT+H_2O_2 cells differentiated spontaneously until day 8 in comparison to their WT counterparts. Genes with statistically significant expression changes are marked in yellow. Selected genes are labeled. Source data are provided as a Source Data file.

H. Enrichment plot from the gene set enrichment analysis (GSEA) of genes differentially expressed between KO (clonal line KO1) vs. WT cells in the RNA-Seq experiment, compared to the reactive oxygen species pathway gene set from hallmark gene sets. We performed 1000 permutations in the GSEA analysis. Gene sets with a false discovery rate (FDR) ≤ 0.057 and enrichment score (ES) ≤ 0.43 are shown.

I. Dot plot illustrates the differential expression of intestine-related genes across the following comparisons: KO vs. WT, WT+H_2O_2 vs. WT, and KO vs. WT+H_2O_2. WT, WT+H_2O_2 and KO cells (clonal line KO1) were differentiated spontaneously until day 8. Genes with higher average expression levels are depicted in red, and those with lower expression levels are shown in blue. Dot size corresponds to the $-\log_{10}(p\text{-value})$.

J. The profile of chromatin accessible signal enrichment around TSS (± 3 Kbp) for WT and KO (clonal line KO2) cells differentiated spontaneously until day 8.

K. Violin plots show peak length distribution in WT and KO (clonal line KO2) cells differentiated spontaneously until day 8. N = indicated number of peaks detected for each sample.

L. Percentage of overlap between peaks and genomic features.

M. PCA of ATAC-Seq data from WT and KO (clonal line KO2) cells differentiated spontaneously until day 8.

N. ATAC-Seq tracks highlight the *loci* of *SOX2* and *DCLK1* in WT (orange) and KO (clonal line KO2, blue) cells differentiated spontaneously until day 8. The peaks represent normalized and combined biological replicates ($N = 2$). Source data are provided as a Source Data file.

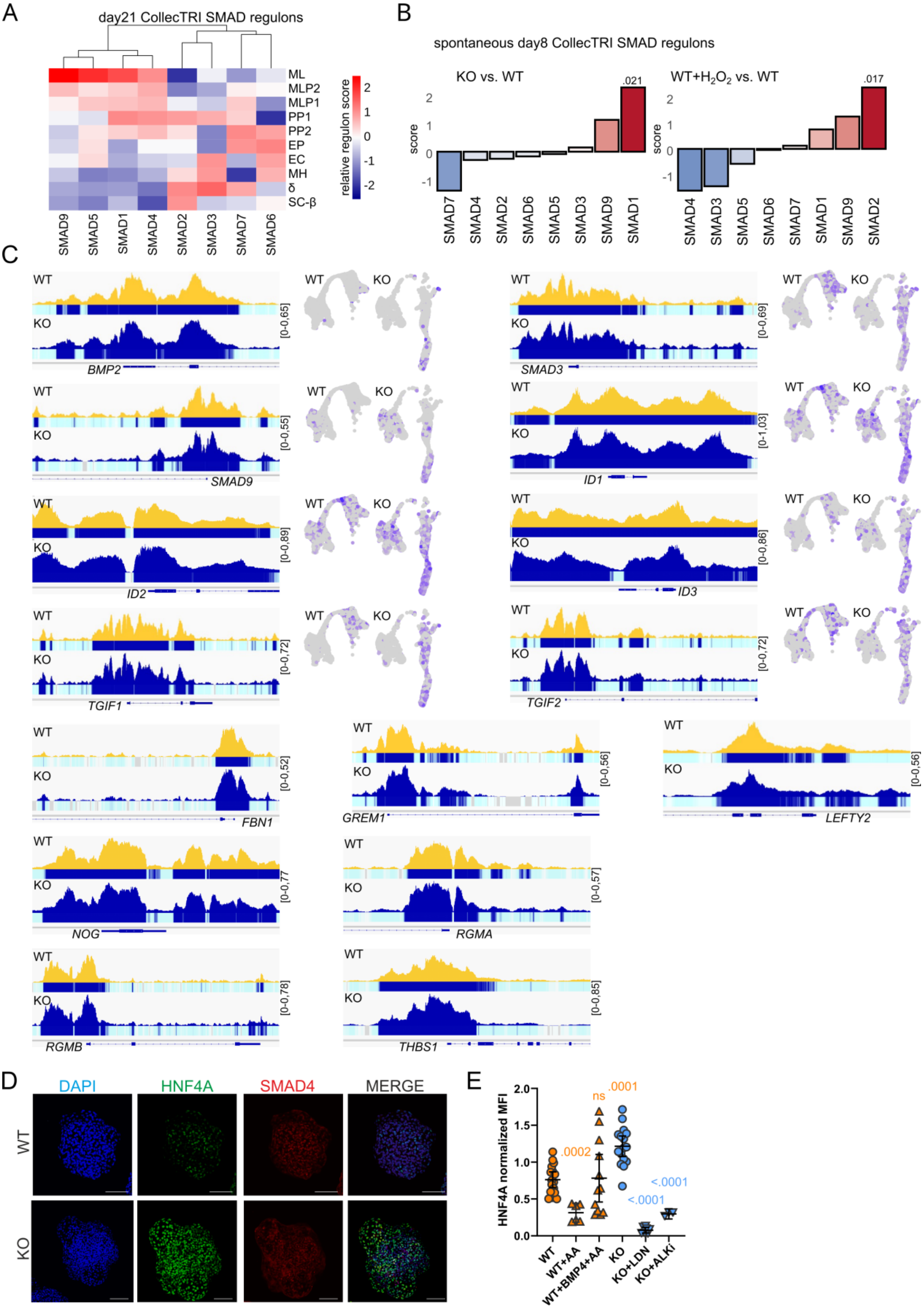

A
day21 CollecTRI SMAD regulons
ML
MLP2
MLP1
PP1
PP2
EP
EC
MH
δ
SC-β
relative regulon score
SMAD9 SMAD5 SMAD1 SMAD4 SMAD2 SMAD3 SMAD7 SMAD6
B
spontaneous day8 CollecTRI SMAD regulons
KO vs. WT
WT+H2O2 vs. WT
score
.021
.017
SMAD7 SMAD4 SMAD2 SMAD6 SMAD5 SMAD3 SMAD9 SMAD1
SMAD4 SMAD3 SMAD5 SMAD6 SMAD7 SMAD1 SMAD9 SMAD2
C
WT
KO
BMP2
SMAD3
SMAD9
ID1
ID2
ID3
TGIF1
TGIF2
FBN1
GREM1
LEFTY2
NOG
RGMA
RGMB
THBS1
[0-0,65]
[0-0,69]
[0-0,55]
[0-1,03]
[0-0,89]
[0-0,86]
[0-0,72]
[0-0,52]
[0-0,56]
[0-0,77]
[0-0,57]
[0-0,78]
[0-0,85]
D
DAPI
HNF4A
SMAD4
MERGE
WT
KO
E
HNF4A normalized MFI
.0002
ns
.0001
<.0001
<.0001
WT
WT+AA
WT+BMP4+AA
KO
KO+LDN
KO+ALKi

Supplementary Figure 8. TGF-β and BMP pathways: analysis of SMAD regulon activity, chromatin accessibility and the influence on the differentiation process

A. Heatmap shows SMAD regulon activity across 10 clusters at the SC-β cell stage on differentiation day 21. Regulons with higher relative activity are depicted in red, and those with lower relative activity are shown in blue. CollecTRI was used for regulon analysis. Source data are provided as a Source Data file.

B. Bar plot shows SMAD regulon activity in KO (clonal line KO1) vs. WT and WT+H_2O_2 vs. WT. Regulons with higher relative activity are depicted in red, while those with lower relative activity are shown in blue. CollecTRI was used for regulon analysis. Source data are provided as a Source Data file.

C. ATAC-Seq tracks highlight the *loci* of selected genes related to TGF-β and BMP pathways in cells differentiated spontaneously until day 8. WT is marked in orange, and KO (clonal line KO2) in blue. The peaks represent normalized and combined biological replicates ($N = 2$). UMAP representation of gene expression at the SC-β cell stage is shown for genes expressed at this stage. Source data are provided as a Source Data file.

D. Representative images of WT and KO (clonal line KO1) cells differentiated spontaneously until day 8, co-stained for HNF4A (green) and SMAD4 (red). DAPI marks nuclei (blue). Scale bar = 100 µm.

E. Dot plot shows quantification of HNF4A immunofluorescence signal in WT (orange) and KO (clonal line KO1, blue) cells treated with activators or inhibitors of the TGF-β and BMP pathways, respectively. Each dot represents one image, with mean and 95% confidence intervals (CIs) indicated. N = 3 independent experiments. An unpaired, two-sided t-test was used to determine statistical significance. Source data are provided as a Source Data file.

sgRNA	sgRNA sequence 5’->3’ (T7-gRNA-TracR)
1	TAATACGACTCACTATAGGGTTGTTCAGTCTGTTAATAGGTGTTTTAGAGCTAGAAATAGCAAGTTAAAATAAGGCTAGTCCGTTATCAACTTGAAAAAGTGGCACCGAGTCGGTGCTTTT
2	TAATACGACTCACTATAGGGTTCTACTATATTACTCCATGTGTTTTAGAGCTAGAAATAGCAAGTTAAAATAAGGCTAGTCCGTTATCAACTTGAAAAAGTGGCACCGAGTCGGTGCTTTT
3	TAATACGACTCACTATAGGGCAAGTTGAATCACCAACCAGTGTTTTAGAGCTAGAAATAGCAAGTTAAAATAAGGCTAGTCCGTTATCAACTTGAAAAAGTGGCACCGAGTCGGTGCTTTT

Supplementary Table 1. sgRNA sequences used for *GPX2* targeting

**Antibody**	**Company**	**Catalog**	**Dilution**	**Diluted in**	**Incubation**
GPX2	Abcam	ab137431	1:1,000	1% BSA	O/N
GPX2	GeneTex	GTX100292	1: 500	5% non-fat dry milk	O/N
GAPDH	Merck Millipore	MAB374	1:2,000	5% non-fat dry milk	O/N
p-SMAD1/5	Cell Signaling	9516T	1:1,000	3% BSA	O/N
FLAG	Sigma	F1804	1:1,000	1% BSA	O/N
anti-rabbit	Sigma Aldrich	A9169	1:5,000	1% BSA	2 h
anti-mouse	Sigma Aldrich	A9044	1:20,000	5% non-fat dry milk	2 h
O/N=overnight					

Supplementary Table 2. Antibodies used for western blot analysis

Antigen	Company	Catalog	Dilution	Primary / Secondary	Host	Reactivity
AFP	SCBT	sc-8399	1:100	Primary	Mouse	Hu, Mus, Rat
CDX2	Abcam	ab76541	1:500	Primary	Rabbit	Hu, Mus, Rat
CHGA	Abcam	ab15160	1:200	Primary	Rabbit	Hu
COL4A	SCBT	sc-59814	1:300	Primary	Mouse	Hu
C-PEP	DSHB	GN-ID4	1:40	Primary	Rat	Hu, Monkey
FLAG	Sigma	F1804	1:500	Primary	Mouse	
FGB	SCBT	sc-271035	1:100	Primary	Mouse	Hu, Mus, Rat
FOXA2	R&D Systems	AF2400	1:100	Primary	Goat	Hu
GCG	SCBT	sc-514592	1:100	Primary	Mouse	Hu, Mus, Rat
GPX2	GeneTex	GTX100292	1:100	Primary	Rabbit	Hu, Mus, Rat
HHEX	R&D Systems	MAB83771	1:100	Primary	Rabbit	Hu, Mus, Rat
HNF4A	SCBT	sc-8987	1:100	Primary	Rabbit	Hu, Mus, Rat
LAMA1	SCBT	sc-74418	1:100	Primary	Mouse	Hu

LAMA5	DSHB	P3H9	1:100	Primary	Mouse	Hu
NKX6-1	DSHB	F55A10	1:20	Primary	Mouse	Hu, Mus
OCT3/4	SCBT	sc-5279	1:50	Primary	Mouse	Hu, Mus, Rat
PDX1	R&D Systems	AF2419	1:100	Primary	Goat	Hu
p-SMAD1/5	Cell Signaling	9516T	1:100	Primary	Rabbit	Hu, Mus, Rat
SMAD2/3	SCBT	sc-133098	1:100	Primary	Mouse	Hu, Mus, Rat
SMAD4	SCBT	sc-7966	1:100	Primary	Mouse	Hu, Mus, Rat
SOX17	R&D Systems	MAB20851	1:400	Primary	Rabbit	Hu
SOX9	Millipore	AB5535	1:300	Primary	Rabbit	Hu, Mus, Rat, Ch
VTN	SCBT	sc-74484	1:100	Primary	Mouse	Hu, Mus, Rat
AlexaFluor 488	Jackson Immunoresearch	715-545-147	1:400	Secondary	Donkey	anti-Goat IgG (H+L)
AlexaFluor 488	Jackson Immunoresearch	711-545-152	1:400	Secondary	Donkey	Anti-Rabbit IgG (H+L)
AlexaFluor 488	Jackson Immunoresearch	705-545-150	1:400	Secondary	Donkey	anti-Mouse IgG (H+L)
TRITC	Jackson Immunoresearch	705-025-147	1:400	Secondary	Donkey	anti-Goat IgG (H+L)

TRITC	Jackson Immunoresearch	715-025-150	1:400	Secondary	Donkey	anti-Mouse IgG (H+L)
TRITC	Jackson Immunoresearch	711-025-152	1:400	Secondary	Donkey	anti-Rabbit IgG (H+L)
AlexaFluor 647	Jackson Immunoresearch	705-605-147	1:400	Secondary	Donkey	anti-Goat IgG (H+L)
AlexaFluor 647	Jackson Immunoresearch	711-605-152	1:400	Secondary	Donkey	anti-Rabbit IgG (H+L)
AlexaFluor 647	Jackson Immunoresearch	715-605-151	1:400	Secondary	Donkey	anti-Mouse IgG (H+L)
Hu – human, Mus – mouse, Ch – chicken						

Supplementary Table 3. Antibodies used for immunofluorescence staining

Antigen	Company	Catalog	Dilution	Primary/ Secondary	Host	Reactivity
CHGA	SCBT	sc-393941	1:300	Primary	Mouse	Hu, Mus, Rat
FGB	SCBT	sc-271035	1:500	Primary	Mouse	Hu, Mus, Rat
INS	Cell Signaling	3014	1:300	Primary	Rabbit	Hu, Mus, Rat
NKX6-1	DSHB	F55A10	1:300	Primary	Mouse	Hu, Mus
OCT3/4	SCBT	sc-5279	1:500	Primary	Mouse	Hu, Mus, Rat
PDX1	R&D Systems	AF2419	1:500	Primary	Goat	Hu
SOX9	Millipore	AB5535	1:500	Primary	Rabbit	Hu, Mus, Rat, Ch
SOX17	R&D Systems	MAB20851	1:500	Primary	Rabbit	Hu

AlexaFluor 488	Jackson Immunoresearch	711-545-152	1:10,000	Secondary	Donkey	Anti-Rabbit IgG (H+L)
AlexaFluor 488	Jackson Immunoresearch	705-545-150	1:10,000 or 1:5,000	Secondary	Donkey	anti-Mouse IgG (H+L)
AlexaFluor 647	Jackson Immunoresearch	705-605-147	1:10,000	Secondary	Donkey	anti-Goat IgG (H+L)
AlexaFluor 647	Jackson Immunoresearch	711-605-152	1:10,000	Secondary	Donkey	anti-Rabbit IgG (H+L)
AlexaFluor 647	Jackson Immunoresearch	715-605-151	1:10,000	Secondary	Donkey	anti-Mouse IgG (H+L)
Hu – human, Mus – mouse, Ch - chicken						

Supplementary Table 4. Antibodies used for flow cytometry

References

1. Ma, Z. *et al.* Deciphering early human pancreas development at the single-cell level. *Nat Commun* **14**, 5354 (2023).
