# Supplementary material for: Oxidative stress and GPX2 control pancreatic vs. non-pancreatic cell fate in human endoderm
Source: Nat Commun. 2026 Jan 3;17:1407. doi: 10.1038/s41467-025-68145-x (PMC12881361; doi:10.1038/s41467-025-68145-x)
Supplement: Supplementary file 2 — Description of Additional Supplementary Files [file 41467_2025_68145_MOESM2_ESM.pdf]

## Description of Additional Supplementary Files

**File Name:** Supplementary Data 1

**Description:** Day 6 bulk RNA-Seq DEGs between *GPX2* KO and WT cells, related to Fig. 3C. Differential expression was assessed using the DESeq2 Wald test (two-sided), and *p*-values were adjusted for multiple comparisons using the Benjamini–Hochberg false discovery rate (FDR) method.

**File Name:** Supplementary Data 2

**Description:** Day 8 scRNA-Seq DEGs between clusters, genotypes within clusters and differential PROGENy pathways analysis, related to Fig. 3G and Supplementary Fig. 4E, G. DEGs were identified using a two-sided Wilcoxon rank-sum test with Benjamini–Hochberg FDR adjustment.

**File Name:** Supplementary Data 3

**Description:** Day 21 scRNA-Seq DEGs between clusters, KEGG pathway analysis, differential PROGENy pathways and CollecTRI regulon analysis, related to Supplementary Fig. 4H, 7A-B, and Supplementary Fig. 8A. Differential expression between clusters was assessed with a two-sided non-parametric test and Benjamini–Hochberg FDR correction.

**File Name:** Supplementary Data 4

**Description:** Differential analysis of genes and CollecTRI regulons from definitive endoderm spontaneously differentiated WT, *GPX2* KO, and WT+$H_2O_2$ cells, related to Fig. 5I, 6F, 7D, and Supplementary Fig. 8B. Differences in gene expression between samples were calculated using a two-sided empirical Bayes moderated t-test (limma-voom), with p-values adjusted for multiple testing using the Benjamini–Hochberg false discovery rate (FDR) method.

**File Name:** Supplementary Data 5

**Description:** Annotated peaks and enriched motifs in definitive endoderm spontaneously differentiated WT and *GPX2* KO cells, related to Fig. 5K-L, 6G, 7E, and Supplementary Fig. 7H-K, 7M, 8C. ATAC-seq peaks were annotated using HOMER.
